# Film-trigger applicator (FTA) for improved skin penetration of microneedle using punching force of carboxymethyl cellulose film acting as a microneedle applicator

**DOI:** 10.1186/s40824-022-00302-5

**Published:** 2022-10-05

**Authors:** Youseong Kim, Hye Su Min, Jiwoo Shin, Jeehye Nam, Geonwoo Kang, Jeeho Sim, Huisuk Yang, Hyungil Jung

**Affiliations:** 1 Department of Biotechnology, Yonsei University, 50 Yonsei-ro, Seodaemun-gu, Seoul, 03722 Korea; 2 Juvic Inc, 208Ho, 272, Digital-ro, Guro-gu, Seoul, 08389 Republic of Korea

**Keywords:** Dissolving microneedle, Carbohydrate polymer film, Insulin, Transdermal drug delivery, Microneedle applicator

## Abstract

**Background:**

Dissolving microneedle (DMN) is a transdermal drug delivery system that creates pore in the skin and directly deliver drug through the pore channel. DMN is considered as one of the promising system alternatives to injection because it is minimally invasive and free from needle-related issues. However, traditional DMN patch system has limitations of incomplete insertion and need of complex external devices. Here, we designed film-trigger applicator (FTA) system that successfully delivered DMN inside the skin layers using fracture energy of carboxymethyl cellulose (CMC) film via micropillars. We highlighted advantages of FTA system in DMN delivery compared with DMN patch, including that the film itself can act as DMN applicator.

**Methods:**

FTA system consists of DMNs fabricated on the CMC film, DMN array holder having holes aligned to DMN array, and micropillars prepared using general purpose polystyrene. We analyzed punching force on the film by micropillars until the film puncture point at different CMC film concentrations and micropillar diameters. We also compared drug delivery efficiency using rhodamine B fluorescence diffusion and skin penetration using optical coherence tomography (OCT) of FTA with those of conventional DMN patch. *In vivo* experiments were conducted to evaluate DMN delivery efficiency using C57BL/6 mice and insulin as a model drug.

**Results:**

FTA system showed enhanced delivery efficiency compared with that of the existing DMN patch system. We concluded CMC film as a successful DMN applicator as it showed enhanced DMN penetration in OCT and rhodamine B diffusion studies. Further, we applied FTA on shaved mouse dorsal skin and observed successful skin penetration. The FTA group showed higher level of plasma insulin *in vivo* than that of the DMN patch group.

**Conclusions:**

FTA system consisting of simple polymer film and micropillars showed enhanced DMN delivery than that of the existing DMN patch system. Because FTA works with simple finger force without sticky patch and external devices, FTA is a novel and promising platform to overcome the limitations of conventional microneedle patch delivery system; we suggest FTA as a next generation applicator for microneedle application in the future.

**Supplementary Information:**

The online version contains supplementary material available at 10.1186/s40824-022-00302-5.

## Background

Recently, oral and hypodermic administration routes are the most widely recognized for successful drug delivery [1, 2]. Oral drug delivery route is most convenient way for patients; however, it has limitation of first-pass effect [3]. In other words, only limited number of oral drugs can retain their efficacy while passing through the gastro-intestinal tract due to hepatic drug metabolism. In contrast, hypodermic route has an advantage that it allows drugs to be directly injected into the body. However, hypodermic injection can cause acute pain to patient and therefore, demands aid of medical expert during administration [4]. Furthermore, injectable routes generate needle waste, which causes other medical hazards such as contaminations, injuries, and infections. Transdermal drug delivery has been developed as an alternative to the existing drug delivery routes in the point that it avoids needle issues and user compliance may be improved by its convenient usability [5].

A dissolving microneedle (DMN), a representative transdermal drug delivery system, has attracted increasing interest as one of the promising types of drug delivery systems. DMN generally consists of drugs and biodegradable and biocompatible polymers appropriate for skin implantation and previous studies suggested carboxymethylcellulose (CMC) and hyaluronic acid (HA) as promising candidates of DMN’s structure polymers [6–9]. DMN delivers drugs after penetration and being dissolved by the interstitial fluid under the skin surface [10]. Since DMN delivery is minimally invasive and painless without generating needle waste [11, 12], it can overcome disadvantages of hypodermic injections such as acute pain, hypertrophic scar, bleeding, and other issues and is considered as a next generation drug delivery system. Therefore, DMN is widely used for delivery of various drug candidates, for example, vaccines, insulin, small molecules, and steroids.

Although DMN has potential of a promising drug delivery platform, it has limitation related to its administration method. DMN is generally administered as a sticky patch containing drug and polymer, which needs to be adhered to the skin after penetration until dissolved by the interstitial fluid [13]. Although application of patch by finger force is convenient, DMN patch system has a limitation of incomplete insertion into skin even when applied using external applicator devices. The basal compartment of DMN does not show effective penetration into the skin after patch administration [14, 15]. Moreover, the applied finger force does not evenly distribute on DMN patch so it is hard to make the DMN successfully penetrate skin [16]. If the microneedle is not sufficiently penetrated into the skin, it may fail to deliver a fixed dose of drug after DMN administration because the DMN is fabricated with drug and polymer. To solve this problem, researchers introduced tip loading DMN patch system [17]. Tip loading DMN is a well-designed microneedle system that can overcome the disadvantage of incomplete insertion of the basal compartment of DMN by loading drug only in the tip compartment of DMN. However, since this system has difficulty in utilizing the basal compartment’s volume, it needs an additional drug concentration condensation step or multiple numbers of DMN in an array to satisfy the drug dose. Thus, insertion of DMN need to be improved to achieve effective drug delivery.

To overcome the limitation of insertion of microneedle patch, researchers have developed various applicators to enhance insertion of DMN patches [18–20]. Generally, a patch applicator consists of an even plate to press the patch and external power source to provide a sufficient force to the plate [21]. Because patch applicators are designed to press the back of DMN patch with plate, the researchers utilized either physical force of compressed spring [22–24] or pressing force of machine [25, 26]. Although these patch applicators allow relatively even distribution of force to DMN patch and improved DMN insertion rate than when administration using finger force, inevitable use of external power source makes this system more complex. Also, these complex external devices attenuate the advantages of a simple patch system that requires only finger force. In addition, sticky patch is still required for the DMN to attach to the skin until it is sufficiently dissolved by the interstitial fluid. Collectively, an ideal applicator should work with simple finger force and avoid complex system and sticky patch.

In this study, we introduce novel patchless DMN application system called film-trigger applicator (FTA). In this system, DMNs are fabricated on the surface of CMC polymer film and micropillars are aligned to the bottom of each DMN. In FTA, DMNs are shot to penetrate the skin without using sticky patch and complex external devices. Further, the FTA system uses an accumulated energy of finger force on the carbohydrate polymer film *via *micropillars, and the film acts like a trigger of a gun at a film puncture point. Here, we used physical property of the film; it breaks at the limit punching force when the film accumulates sufficient energy by micropillars, FTA operates with finger force and is different from existing applicators that use external energy sources. We analyzed physical properties of the CMC polymer film and performed *in vitro* skin penetration test using different concentrations of CMC and micropillar diameters to evaluate this novel system. Moreover, we designed *in vitro* experiments, including optical coherence tomography (OCT) and fluorescent dye diffusion in cadaver skin, for further evaluation. The results of OCT ascertained improved physical penetration and fluorescence data showed superior drug diffusion of the FTA system compared with that of conventional DMN patch application. Furthermore, we used insulin as target drug for *in vivo* experiments to show efficient drug delivery of the FTA system and observed enhanced insulin delivery by FTA using only finger force without any external force source compared with that of the existing microneedle patch delivery systems.

## Methods

### Materials

CMC (90 kDa) and human insulin were purchased from Sigma-Aldrich (St. Louis, MO, USA). HA (30 kDa) was purchased from Uscarepharm (Suwon, Republic of Korea). Sterile microtube (2.0 mL) was purchased from Anxygen Inc. (Union city, CA, USA). Human insulin ELISA kit ((80-INSHU-E01.1) was purchased from ALPCO (Salem, NH, USA). Blood glucose detection kit (ACCU-CHEK) was purchased from Roche (Basel, Switzerland).

### Preparation of CMC polymer film, Micropillar and array holder

We selected CMC as the polymer film material because it is biodegradable and biocompatible carbohydrate polymer widely used for microneedle fabrication [27–29]. CMC powder was dissolved in distilled water to prepare solutions of 2% (w/v), 4% (w/v), and 6% (w/v) for fabricating CMC films of square shape on stainless-steel frame. The metal frames were subjected to vacuum plasma treatment to increase polymer attraction on the frame; 300 μL CMC solution of each concentration was poured on the square frame of size 2.5 × 2.5 cm^2^. The solution was dried for 12 h at room temperature (25 °C) to obtain CMC film.

We firstly made metallic mold for duplicating general purpose polystyrene micropillars and array holders. The general purpose polystyrene which has better price, disposable usage and superior mass production potential than metallic micropillars was adapted for FTA material. We utilized customized laser cutting machine from MECHA-4U (Hwaseong, Republic of Korea) to carve the metallic mold for micro-scale pillar design. Next, we poured melted general purpose polystyrene inside the mold and we took micropillars and array holders out of the mold after solidification.

### Mechanical properties of polymer film for FTA application

Mechanical properties of CMC film were analyzed using a force analyzer (Z0.5TN; Zwick/Roell Inc., Ulm, Germany). For the puncture test, we recorded the axial force applied on the film by keeping the sensor on micropillars of the film placed in vertical downward direction. The test was performed five times for each experimental group. The following equations were used for calculating the punching force required for the puncture test:

1 $$F=\upsigma \pi DT$$

where F is the punching force (N), which is calculated by multiplying crack length same as the perimeter of micropillar ($\pi D$; $D$ is the diameter of micropillar; µm), thickness of the film (T; µm), and shear stress of CMC film (σ; N/µm^2^) [30].

2 $${G}_{f }=\frac{{W}_{f}}{A}$$

The above equation [31, 32] was used to calculate fracture energy (${G}_{f}$) required to make puncture on the film by dividing the work done for film puncture (${W}_{f}$; the area under the force–displacement curve) by the film puncture area (A), which is cross-sectional area of micropillars in contact with the film.

3 $${W}_{f}=\int Fds$$

where ${W}_{f}$ represents work, F is applied force and s is displacement length in line with force [31]. In our experiments, the displacement length represents the distance moved by the sensor after film contact.

### DMN fabrication and morphology

DMNs were fabricated in candlelit microneedle (CMN) conformation following two steps of fabrication of base layer and head layer [13]. HA aqueous solution (20% w/v) droplets were dispensed on the CMC film and placed in contact with a cover hydrophobic plate. A sandglass shape was obtained by vacuum drying with a constant distance of 300 µm between the two plates. Subsequently, HA aqueous solution (60% w/v) droplets were dispensed on sandglass structures and subjected to centrifugal lithography at 202 g force for 1 min. All DMN solutions were loaded with 0.1 IU insulin per 25 microneedles [33, 34]. DMNs were fabricated using cold jacket and stored at 4 °C. Morphological conformation of the fabricated microneedles was observed using a bright field microscope (M165 FC; Leica, Wetzlar, Germany) and surface of DMNs was analyzed using scanning electron microscope (SEM, IT-500HR; JEOL, Tokyo, Japan).

### High-performance liquid chromatography (HPLC) of fabricated insulin loading DMN

We analyzed insulin amount in DMN using reverse-phase HPLC (Waters 600S, Waters, Milford, MA, USA) with a C18 column (150 mm × 4.6 i. d., Cosmosil 5C18-AR-II, Nacalai Tesque Inc., Kyoto, Japan). We set our mobile phase with solution A (aqueous solution of 0.2 M anhydrous sodium sulfate with phosphoric acid; pH 2.3) and solution B (0.1% v/v, trifluoroacetic acid in acetonitrile). We ran the machine with isocratic phase A:B (3:1). The wavelength for detecting insulin was 214 nm and flow rate was 1.0 mL/min. We serially diluted a sample solution of insulin with PBS (from 0 to 500 μg/mL) to prepare a calibration curve to meet R square value ≥ 0.99. The loading amount difference of DMN patch and FTA groups was non-significant.

### Pharmacokinetic analysis of DMN delivery system

Pharmacokinetic were calculated from the plasma insulin concentrations versus time. The maximum drug concentration. The area under the drug concentration versus time after administration curve (AUC) was calculated and we finally determined the bioavailability by following equation below.

$$\%\;\mathrm{bioavailability}\:=\:\lbrack\mathrm{AUC}(\mathrm{DMN})\;/\;\mathrm{AUC}(\mathrm{injection})\rbrack\:\times\:\lbrack\mathrm{injection}\;\mathrm{dose}/\;\mathrm{DMN}\;\mathrm{dose}\rbrack\:\times\:100$$

### Optical coherence tomography

The skin penetration ability of DMN patch and FTA were analyzed by OCT (Kyungpook National University, Daegu, Korea). Shaved pig cadaver skin (approximately 1.0–1.2 mm thickness; Cronex, Hwaseong, Korea) was treated with DMN and its penetration ability was observed by OCT without histological sections.

### Skin permeation analysis using rhodamine B as fluorescent model dye

We applied 25 microneedles (5 × 5 arrays) each to pig cadaver skin using DMN patch and FTA. We conducted *in vitro* skin permeation study by substituting insulin with rhodamine B (Sigma-Aldrich). After 12 h, rhodamine B fluorescence were obtained M165 FC fluorescence microscope (Leica), and fluorescence intensity was calculated using the ImageJ software (National Institutes of Health, Bethesda, MD, USA). We visualized fluorescence diffusion from the bottom view opposite of microneedles penetration site (*n* = 5/group) [35].

### Animals and ethical approval

We followed the ethical guidelines and regulations of the Yonsei Laboratory Animal Research Center (YLARC) for animal experiments. Institutional Animal Care and Use Committee (IACUC, approval number: IACUC-A-202110–1354-02) of the Yonsei University approved all procedures. Female C57BL/6 mice (6-weeks old) were purchased from Orient Bio (Seongnam, Korea) and allowed to adapt for one week. Mice in microneedle administration group were shaved for microneedle insertion. The mice were treated with insulin (0.1 IU) through subcutaneous injection, DMN patch, and FTA (*n* = 5/group). Mice of all groups were kept under fasting condition for 12 h before the experiments. All mice were housed at room temperature (22–24 °C) with a 12 h light/dark cycle.

### Tracing residual DMN after administration of DMN patch and FTA

For tracing dissolution of microneedles in DMN patch, we detached DMN patch from the skin after 5, 10, 15, and 30 min. However, we could not detach microneedles applied by FTA because the microneedles were implanted inside the skin. Thus, we vertically observed the microneedles administered skin by FTA in 5, 10, 15, and 30 min.

### Blood glucose monitoring and plasma insulin detection

In animal experiments, we selected 3 × 3 array DMNs to meet 0.1 IU/array as mice application dose. DMNs loading 0.1 IU insulin were administered to the skin via DMN patch and FTA along with positive control (0.1 IU subcutaneous injection of insulin) and negative control (without treatment). Mice of each group were fasted for 12 h before the experiments. After administration of subcutaneous injection, DMN patch, and FTA, we monitored blood glucose (mg/dL) at 0, 1, 2, 4, 8, and 12 h using blood glucose detector (ACCU-CHEK). We simultaneously collected tail vein blood (100 µL) from mice of all groups including negative control at 0, 2, 4, 8, and 12 h. The collected blood samples were centrifugated in 300 × g for 15 min to obtain supernatant fluids. The plasma insulin concentration (µIU/mL) in supernatant fluid was analyzed by insulin ELISA kit (80-INSHU-E01.1). The number of mice in each experiment was five.

### Statistical analysis

SPSS (IBM, Armonk, NY, USA) was used for statistical analyses. All data were analyzed by one-way analysis of variance (ANOVA) followed by Scheffe post-hoc test or Student’s *t*-test. Statistical significance was set at *p* < 0.05 (^*^p < 0.05, ^**^p < 0.01, ^***^p < 0.001).

## Results

### Preparation of carbohydrate polymer film for FTA system and its force analysis

**Fig. 1:**
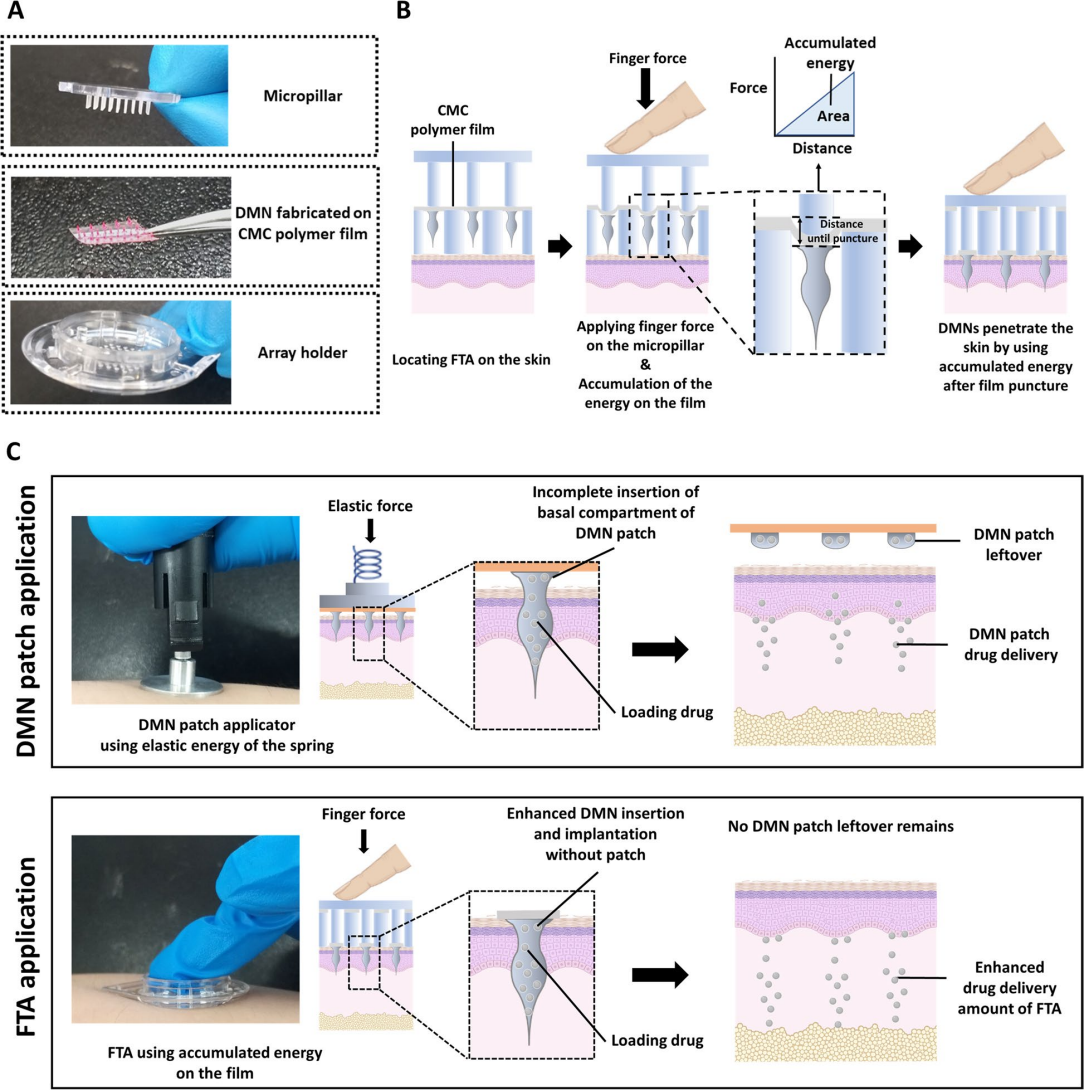
Schematic representation of FTA system. **a** Representative image showing three parts of FTA system. **b** Application steps of FTA system. **c** Comparison between the existing DMN patch applicator and FTA system

FTA is a patchless system of DMN administration, which enables implantation of DMNs inside the skin using carbohydrate polymer film. As shown in Fig. 1a, the FTA system consists of three parts: micropillars, DMNs fabricated on carbohydrate film, and array holder. When a force is applied vertically to the plain of the film, it is accumulated as energy until the film punctures (deformation). In other words, when a force is applied on the film by the micropillars, it changes the distance (length change) of the film until it reaches the puncture resistance. The energy required for film puncture is accumulated until its puncture, and DMNs use this energy to penetrate the skin (Fig. 1b). We utilized the physical properties of the film for DMN penetration; micropillars also play crucial role in applying force on the film until accumulation of sufficient energy.

The FTA system is different from the existing DMN applicators as it operates through applied force on the polymer film (Fig. 1c). The existing DMN applicators control its application force using elastic force of a spring, whereas the FTA system utilizes accumulated finger force applied on the polymer film through micropillars. The existing DMN patch application system has a limitation of residual DMN patch after detaching from the skin, whereas the FTA system enables DMN insertion without sticky patch and with relatively enhanced drug delivery compared with that of the existing DMN patch.

**Fig. 2:**
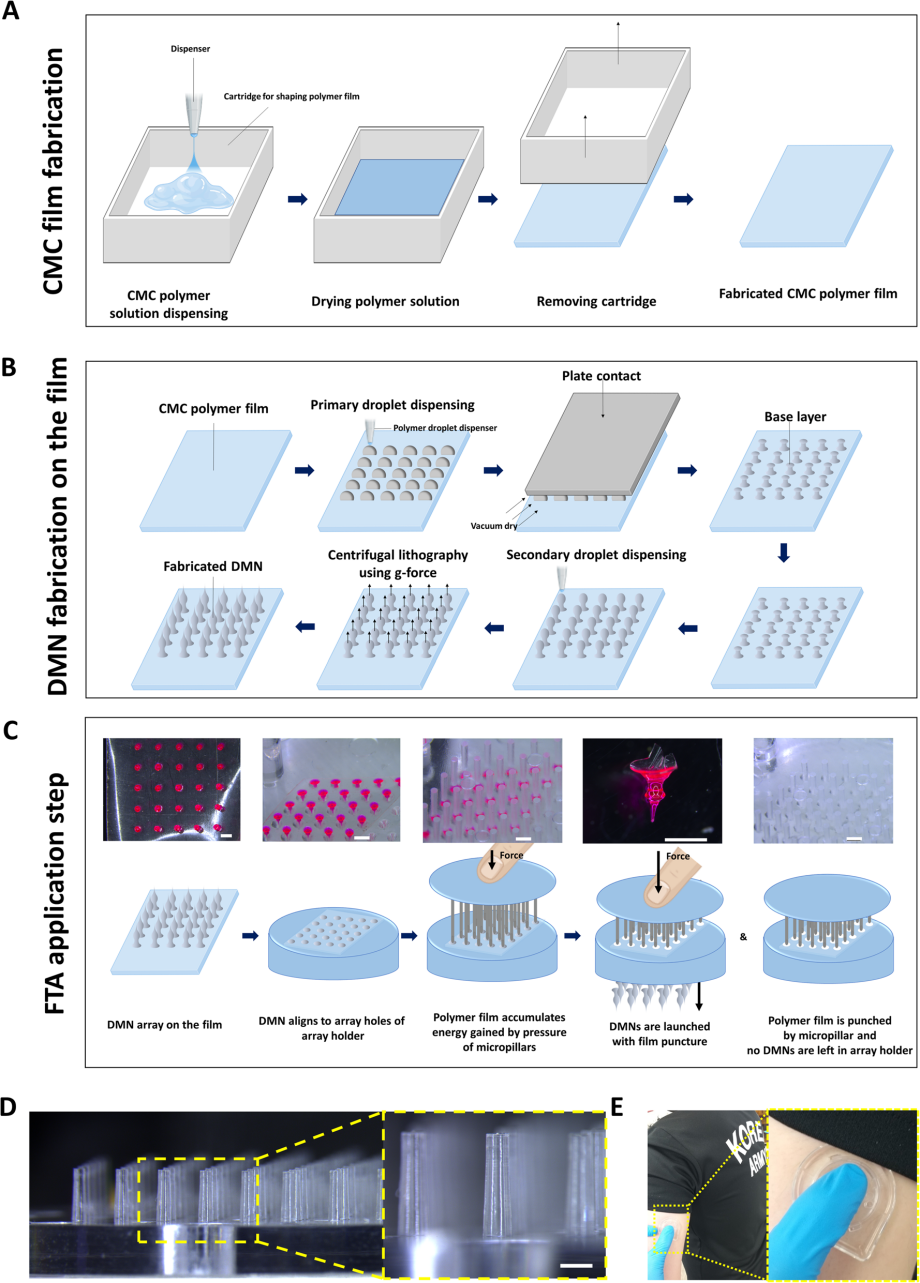
**a** Schematic steps for fabrication of carbohydrate polymer CMC film. **b** Schematic steps for fabrication of DMN on CMC film. **c** Light microscope and schematic images of FTA application steps (Scale bar, 500 µm). **d** Light microscope image of general purpose polystyrene micropillars (Scale bar, 500 µm). **e** Example of expected human application site for FTA

To explain our FTA concept, fabrication of the film from CMC polymer solution, DMN fabrication on the film, and application steps are illustrated in Fig. 2. We fabricated the CMC polymer films (Fig. 2a) by drying the polymer solution. Next, we fabricated DMNs on the film by centrifugal lithography [36] in CMN conformation (Fig. 2b) [37]. For DMN fabrication, we dispensed HA droplets on the film, which were dried by contacting a hydrophobic plate to obtain sandglass-like shape initially. We then dispensed secondary droplets on the sandglass structures and fabricated DMN by centrifugal force (202 g) using a customized rotor.

The application steps of FTA works are described in Fig. 2c. First, each DMN from the array was aligned on the holes of the FTA array holder (the holes in array holder were designed to match DMNs in array). Next, the micropillars applied force on the film, which was accumulated as energy and punctured the film when it reaches the limit of puncture resistance. At the moment of film puncture, the micropillars pushed DMNs through the holes (diameter = 500 µm) using accumulated energy until the puncture resistance, and DMNs penetrated the skin. The diameter and height of micropillar were 350 µm and 1.5 mm, respectively (Fig. 2d). The usage example of the FTA described in Fig. 2e. The micropillar’s diameter was designed smaller than the bottom diameter of DMN (421 ± 32 µm) (see Additional file 1).

**Fig. 3:**
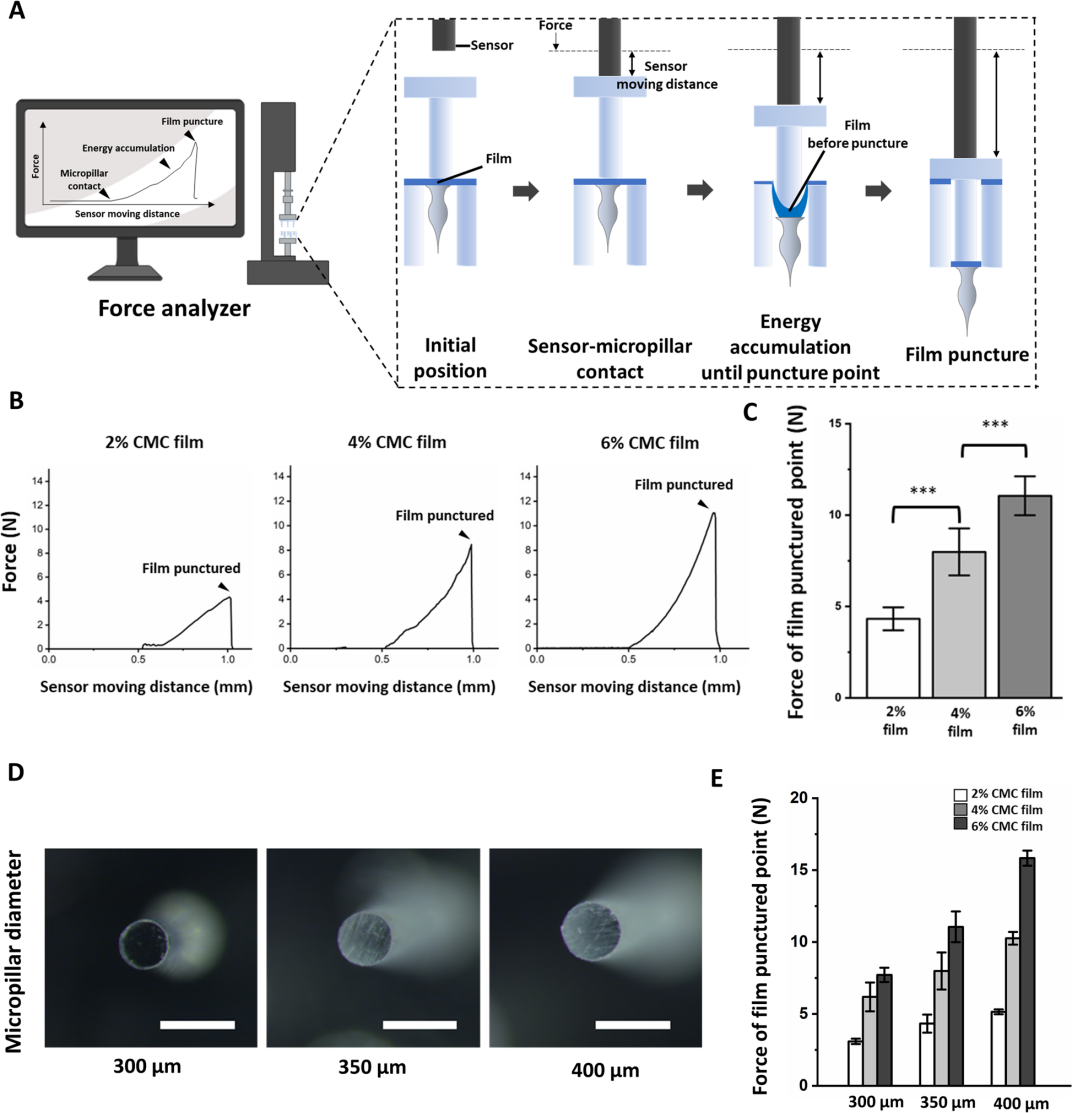
**a** Schematic observation of accumulated energy on the polymer film and puncture force using a force analyzer. Actual analysis was conducted using 5 × 5 DMN FTA array. **b** Representative image of accumulated energy on the CMC film by the pressure of micropillars (350 µm diameter) until the film puncture point at different CMC film concentrations. **c** Applied force at film puncture of 350 µm diameter micropillar according to the film’s polymer solution concentration. (*n* = 5, mean ± S.E.). **d** Image showing 300 µm, 350 µm, and 400 µm diameter micropillars used in the experiment (Scale bar = 500 µm). **e** Measured film puncture force of 2%, 4%, and 6% CMC film following the micropillars’ diameter (300 µm, 350 µm, and 400 µm) (*n* = 5, mean ± S.E.). Statistical significance was set at *p* < 0.05; ^*^p < 0.05, ^**^p < 0.01, ^***^p < 0.001

To identify mechanism of FTA application, we analyzed the force values applied on CMC films until film puncture using 2%, 4%, and 6% CMC film. The principal of force analyzer is illustrated in Fig. 3a. We aligned micropillars on the DMN array, and the sensor of the force analyzer was moved toward the micropillars. When the sensor came in contact with the micropillars, it started to push the micropillars, and the force value increased until film puncture. The distance moved by the sensor after the sensor contacts with the micropillar for each film concentration was approximately 0.5 mm, and it is inferred that the area beneath the force-distance graph may be translated as energy accumulated on the film (Fig. 3b) using Eq. (3) presented in Methods section. Finally, the film was punctured and DMNs on the film were shot similar to pulling the trigger of a gun. The force value from the force analyzer plunged after film puncture. The value of punching force at film puncture point was gradually increased according to the concentration of CMC films. The values of punching force were 4.35 ± 0.62 N (2%), 8.02 ± 1.29 N (4%), and 11.05 ± 1.07 N (6%) (Fig. 3b & c). We did not conduct experiment with polymer concentration of CMC film more than 6% because it is known that single finger force of ordinary people is approximately 12 N [38].

Furthermore, we measured punching force at film puncture point for each of 2%, 4%, and 6% CMC film at different micropillars’ diameter of 300 µm, 350 µm, and 400 µm (Fig. 3d) and observed that the force required for film puncture had a correlation between force and micropillars’ diameter as shown in Table 1. We observed that the punching force increased with increase in micropillar’s diameter (Fig. 3e). The film puncture force of 300 µm micropillars for 2%, 4%, and 6% CMC films was 3.10, 6.20, and 7.71 N, respectively, whereas it was 4.32, 7.98, and 11.05 N respectively for 350 µm micropillars, and 5.54, 10.26, and 15.83 N respectively for 400 µm micropillars. To sum up, film punching force showed a tendency to increase with increase in polymer concentration and micropillar diameter.

**Table 1:** Average film puncture force of 2%, 4%, and 6% CMC film with different micropillars’ diameters

**Micropillar diameter**	**300 µm**	**350 µm**	**400 µm**
**2% CMC film**	3.10 N	4.32 N	5.54 N
**4% CMC film**	6.20 N	7.98 N	10.26 N
**6% CMC film**	7.71 N	11.05 N	15.83 N

### Evaluation of DMN penetration of the FTA system using OCT imaging and drug diffusion using rhodamine B model dye

**Fig. 4:**
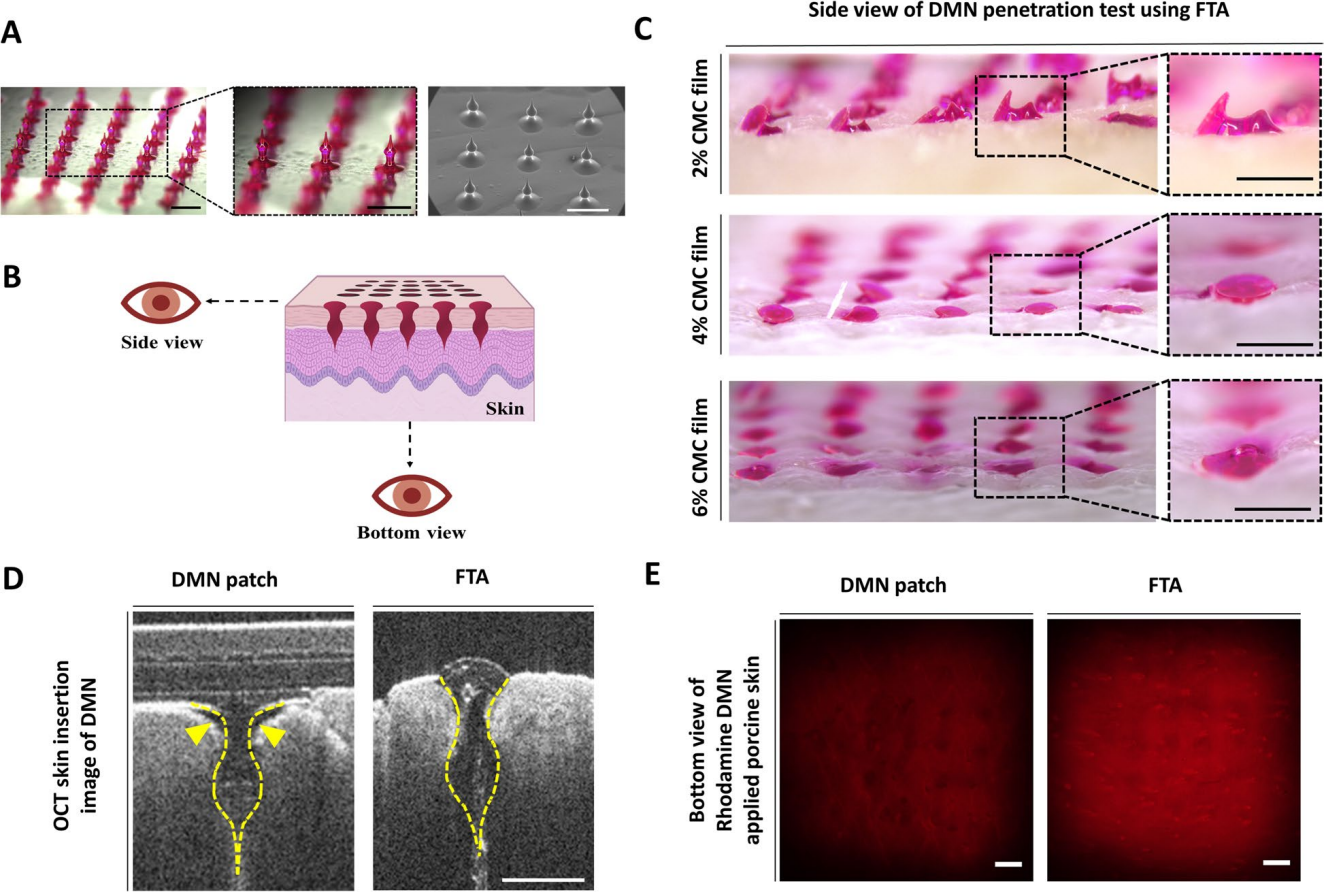
DMN penetration analysis and rhodamine B model drug diffusion test using FTA system *in vitro*. **a** Optical microscope and SEM images of DMN arrays fabricated on the film (Scale bar, 1 mm). **b** View-point instruction of DMN application using FTA. **c** DMN application efficiency of FTA system at different concentrations of CMC films (Scale bar, 500 µm). **d** OCT images of DMN patch and FTA system. Dashed line indicates the shape of DMN inserted in the skin. Arrowhead indicates cavity formed after DMN patch application (Scale bar, 500 µm). **e** The bottom view of fluorescence diffusion of rhodamine B-loaded DMN applied to the porcine skin at 12 h after penetration (Scale bar, 1 mm)

We used rhodamine B as model dye for visualizing delivery efficiency of the FTA system. Figure 4a shows conformation of rhodamine B-loaded DMN obtained using optical microscope and SEM. Optical microscopic image showed that the DMN array had a homogeneous shape and SEM image revealed that DMNs had smooth and fine surface texture. Figure 4b shows the view-point of administered DMN. Interestingly, DMN penetration into pig cadaver skin was different at different CMC film concentrations of the FTA system, as shown in the side view of DMN administration (Fig. 4c). With 2% CMC film, DMN penetration was unsuccessful with force value of 4.35 ± 0.24 N. Surprisingly, when 4% (8.02 ± 0.30 N) and 6% (11.05 ± 0.42 N) CMC films were used, DMN penetration was successful. In particular, 6% CMC film concentration showed more penetration than that of 4% film.

Because OCT shows vertical section of DMN-administered skin [39], we acquired OCT image for observing penetration and insertion of DMN patch and FTA using 6% film that showed the most successful penetration among all experimental groups (Fig. 4d). For comparison, DMN patch was applied using a patch applicator having a round shape plate and designed according to DMN patch size (diameter: 2 cm) to press the back of DMN patch because improved DMN insertion was observed by using a patch applicator compared with that of bare hand application [40]. DMN patch applicator could apply a force of 3.60 kgf (approximately 35.28 N) to the patch for skin insertion. However, in OCT image of DMN patch, we could observe incomplete insertion of basal compartment of DMN (yellow arrowhead) because of its method of patch administration. On the other hand, FTA enabled insertion of bottom compartment of DMN into the skin.

In addition, we administered rhodamine B fluorescent dye-encapsulated DMN to pig cadaver skin through DMN patch and FTA (Fig. 4e). Higher fluorescent intensity was detected in the bottom view because fluorescence diffuses vertically deeper inside pig cadaver skin. We observed greater fluorescence intensity after 12 h in the FTA group (102.52 ± 7.81) than that in the DMN patch group (70.36 ± 3.83); the intensity was described as relative fluorescent intensity analyzed using the ImageJ software (see Additional file 2).

### Insulin delivery efficiency of FTA system in vivo

**Fig. 5:**
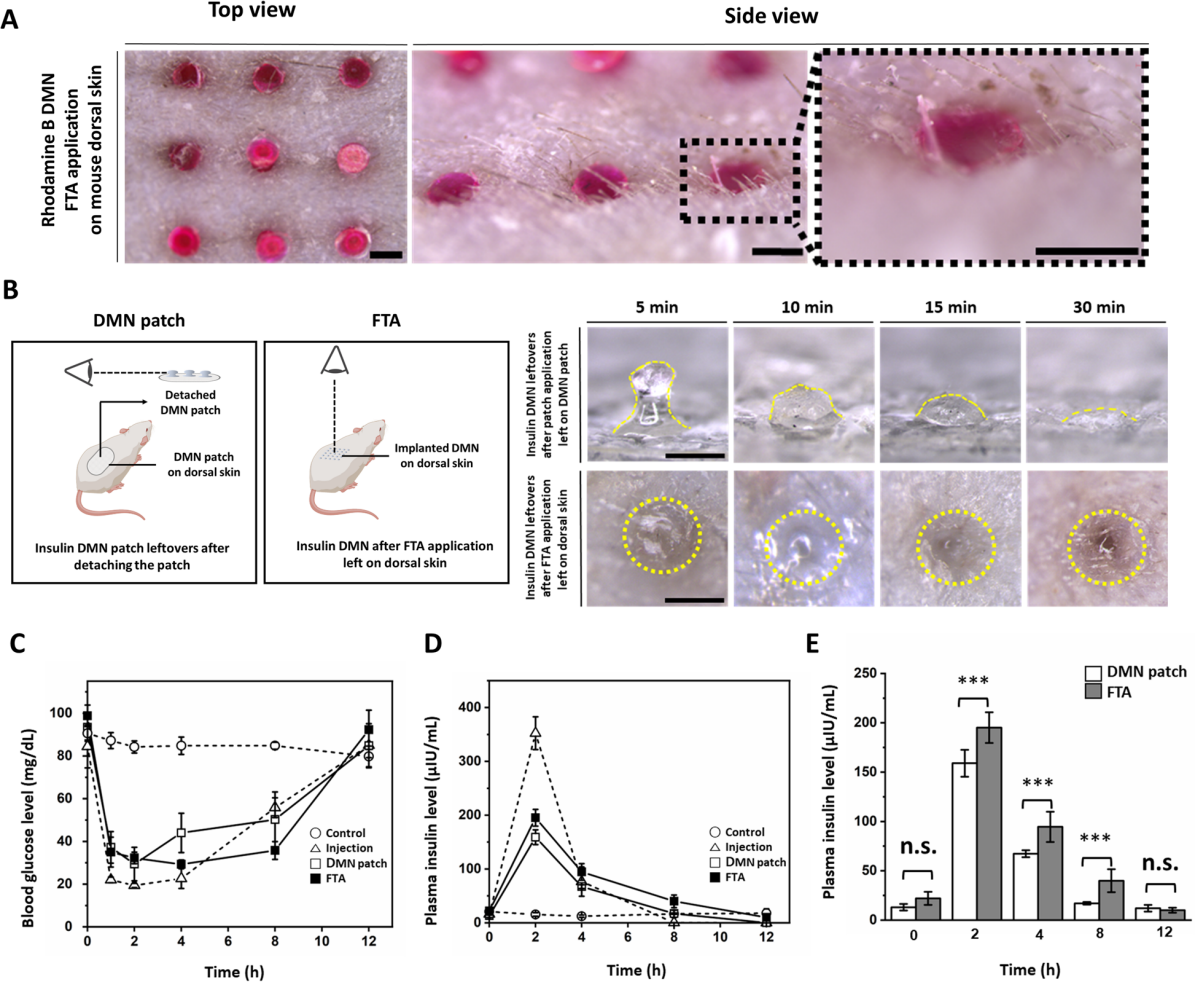
Mouse *in vivo* experiments of FTA system (**a**) Visualization of successful skin insertion using rhodamine B-loaded DMN into mouse skin (Scale bar, 500 µm). **b** Description for the observation concept and dissolution of insulin microneedle leftovers in DMN patch and dissolution of microneedles administered into mouse skin using FTA (Scale bar, 500 µm). **c** Blood glucose level in the control, subcutaneous insulin injection, insulin DMN patch, and insulin DMN FTA groups (*n* = 5, mean ± S.E.). **d** Plasma insulin level of the control, subcutaneous insulin injection, DMN patch, and FTA groups (*n* = 5, mean ± S.E.). **e** Plasma insulin concentration in the DMN patch and FTA groups expressed in a bar graph. Statistical significance was set at *p* < 0.05; ^*^p < 0.05, ^**^p < 0.01, ^***^p < 0.001. n.s. indicates non-significant

We assesses fluorescent model dye delivery efficiency of FTA system *in vitro*. Further, we selected insulin for evaluation of delivery efficiency of FTA *in vivo* because insulin delivery can be easily traced by measuring blood glucose level. Furthermore, we compared the rate of insulin delivery from the FTA system and DMN patch by measuring plasma insulin level. We also confirmed DMN penetration by the FTA system into live animal skin through successful insertion of rhodamine B-loaded DMN by FTA as visualized in Fig. 5a. We showed the top and side view of DMNs inserted into shaved mice skin. We could only detect the bottom view of DMNs, demonstrating that our FTA successfully lead DMNs to penetrate the skin barrier.

Further, we assessed dissolution of inserted insulin DMN patches on mice skin (Fig. 5b) by detaching them from mouse dorsal skin after 5, 10, 15, and 30 min because it is widely known that DMN patches needs approximately 30 min to dissolve [41]. DMN patch’s top view was hard to detect because of sticky patch’s opaque material and DMNs implanted by FTA were not able to detach because those are implanted into skin and quickly turns into gel by interstitial fluids. Thus, we observed DMN dissolution over time on mouse dorsal skin using the method described in Fig. 5b. We first applied DMN patches on live mouse dorsal skin using DMN patch applicator and detached the patches for observing after 5, 10, 15, and 30 min. As a result, we observed some remains in insulin DMN patches shown by yellow dashed line. In contrast, in the FTA experimental group, we could observe only the bottom surface’s conformation of insulin-loaded DMNs until 5 min after insertion. Interestingly, the bottom of insulin DMNs swelled due to interstitial fluid absorption in 10 min after insertion. Finally, insulin DMNs were completely dissolved and absorbed, and we could detect only pore without DMN remains at 30 min after insertion in the FTA group. The yellow dashed circles (Fig. 5b) indicate insulin DMN leftovers after FTA application on mouse dorsal skin.

In *in vivo* experiment, insulin (0.1 IU) was loaded in all experimental groups (subcutaneous injection, DMN patch, and FTA). The blood glucose levels were measured at 0, 1, 2, 4, 8, and 12 h (Fig. 5c, Additional file 3a). Marked decrease in blood glucose level within 1 h (22.00 ± 1.34 mg/dL) along with fast recovery of blood glucose level between 4 h (22.63 ± 4.63 mg/dL) and 8 h (55.81 ± 4.26 mg/dL) was detected in the insulin injection group. Conversely, the blood glucose level in the FTA group (35.01 ± 6.98 mg/dL) within 1 h was higher than that of the injection group, and it showed slower recovery than that of the insulin injection group between 4 h (29.36 ± 2.02 mg/dL) and 8 h (35.80 ± 4.21 mg/dL). In addition, the FTA group delivered higher amount of insulin than that of the DMN patch group because the FTA group showed higher plasma insulin levels that those of the DMN patch group (195.12 ± 15.54 versus 159.01 ± 13.70 µIU/mL, 94.55 ± 15.43 versus 67.33 ± 3.50 µIU/mL, and 39.97 ± 11.70 versus 17.07 ± 1.32 µIU/mL in 2, 4, and 8 h, respectively) (Fig. 5d & e).

## Discussion

### Preparation of CMC polymer film for FTA system and its force analysis

As we described in the Results section, the film punching force was 4.35 ± 0.62 N (2%), 8.02 ± 1.29 N (4%), and 11.05 ± 1.07 N (6%) with 350 µm micropillar diameter, indicating that the film punching force increases with increase in the film’s polymer concentration with same micropillar diameter because the thickness of the film increases at higher CMC concentration in (see Additional file 4). In addition, we observed that the punching force needed for film puncture is proportional to the diameter of micropillars at same film concentration (Table 1). We have shown that the distance moved by the sensor was approximately 0.5 mm in each group with different film polymer concentration; the area under the force-sensor moving distance graph indicates accumulated energy on the film until film puncture at each polymer concentration. The reason for 2% film being unable to penetrate the porcine skin (Fig. 4c) was that the corresponding FTA could not accumulate sufficient energy to insert DMN into the skin. Contrarily, the FTA system with 4% film showed highly improved DMN insertion but was not completely successful. Surprisingly, FTA with 6% CMC film showed penetration of all DMNs in array into the cadaver skin. These results suggest that a punching force of 11 N of 6% film is desirable to effectively administer DMNs into the skin. Thus, we inferred that film thickness determined by film CMC concentration affected the punching force based on the data (Additional file 4, Table 1) and Eq. (1) described in the Methods section.

We also showed a correlation between force and micropillars’ diameter (area) in Table 1. The force needed for film puncture increased with increase in micropillar’s diameter (Fig. 3e). For example, the film puncture force of 6% CMC film, which was selected for in vivo experiment, with 350 µm micropillar diameter (11.05 N) was higher than that with 300 µm micropillar diameter (7.71 N), and it was higher with 400 µm micropillar diameter (15.83 N) than that with 350 µm micropillar diameter (11.05 N). It might be due to greater force being required to make puncture with micropillars having large diameter and area at the same film concentration. This implied that film puncture force is also controlled for FTA application by adjusting micropillars’ diameter and film concentration.

### Evaluation DMN penetration of the FTA system using OCT imaging and drug diffusion using rhodamine B model dye

Figure 4d shows OCT image of DMN patch, which reveals incomplete insertion of DMN (yellow arrowhead) because of its method of patch administration. It might be due to the even plate of DMN patch applicator is not suitable for actual uneven indentation on the skin surface generated by sharp DMNs being pressed against the skin, which leaves space between the patch and skin indicated by yellow arrow-heads in Fig. 4d. However, compared with DMN patch application, FTA application allowed insertion of the most part of DMN into the skin and it might penetrate epidermis layer and reach upper dermis considering the height of DMN (750–800 µm). It is expected that micropillars in FTA individually aid insertion of each single DMN and leave less space because micropillars has markedly narrow area in contact with the skin compared with that of DMN patch applicator. In addition, micropillars have advantage of insertion force; for instance, DMN patch applicator could apply a force of 3.6 kgf (approximately 35.28 N), however, it had difficulty in DMN skin insertion compared with that of FTA with 6% CMC film that could apply a force of approximately 11 N. These results are due to difference in the area of contact with the skin. DMN patch applicator had 1π cm^2^ of plate area, which was approximately 33-fold of 0.03π cm^2^ of 25 micropillars’ area. Thus, FTA with narrow area of contact with the skin caused by micropillar may be less likely to meet uneven skin surface causing development of space between the skin and DMNs. Further, according to the Eq. (2) described in the Methods section, extremely smaller cross-sectional area is advantageous for DMN skin penetration because more energy per unit area may be transferred with the smaller cross-sectional area under same force value. Thus, we ascertained that FTA successfully inserted DMNs into the skin with proper CMC film concentration.

Additionally, Fig. 4d and e showed that FTA inserted DMNs into deeper skin layers, affecting deeper drug diffusion into the skin. We administered rhodamine B-loaded DMN into pig cadaver skin using DMN patch and FTA. We detected stronger fluorescence intensity of rhodamine B in the bottom view due to diffusion of rhodamine B deeper inside the cadaver skin. Thus, we observed fluorescence in the bottom view of pig cadaver skin, which implied vertical drug diffusion, and it was obtained 12 h after DMN skin penetration because it was needed to confirm the delivery pattern after sufficient diffusion time had elapsed. Moreover, we detected higher fluorescence intensity in the FTA group (102.52 ± 7.81) than that in the DMN patch group (70.36 ± 3.83) (see Additional file 2).

### Insulin delivery efficiency of FTA system in vivo

We measured blood glucose levels at 0, 1, 2, 4, 8, and 12 h (Fig. 5c, Additional file 3a). The results implied that FTA was able to decrease blood glucose level continuously and slowly until 8 h, in contrast to insulin injection that showed marked decrease in blood glucose level until 2 h and fast recovery of blood glucose level between 4 and 8 h. These results explained that whether delivery routes were affected by the skin barrier or not. In other words, the subcutaneous injection route rapidly delivered drugs inside the body without disturbance of skin barrier, whereas insulin encapsulated in DMN was released at a slower rate because of the hinderance of the skin barrier to diffusion of drug [42].

Interestingly, we observed that FTA showed enhanced insulin DMN delivery efficiency than that of the existing DMN patch system as the FTA group showed lower levels of blood glucose than those of the DMN patch group (29.36 ± 2.02 mg/dL versus 44.08 ± 9.24 mg/dL and 35.80 ± 4.21 mg/dL versus 50.25 ± 13.09 mg/dL in 4 and 8 h, respectively) (Fig. 5c, Additional file 3a). This result matched with higher levels of plasma insulin in the FTA group than those in the DMN patch group (195.12 ± 15.54 µIU/mL versus 159.01 ± 13.70 µIU/mL [approximately 1.23-fold], 94.55 ± 15.43 µIU/mL versus 67.33 ± 3.50 µIU/mL [1.41-fold], and 39.97 ± 11.70 µIU/mL versus 17.07 ± 1.32 µIU/mL [2.35-fold] in 2, 4, and 8 h, respectively) (Fig. 5d & e, Additional file 3b); the difference in insulin level might be due to DMN remains in patch application (Fig. 5b). In further, Fig. 5D and E indicate plasma insulin concentration versus time after dosage of mice and we calculated area under the curve (AUC) values of FTA and DMN patch groups when the injection group’s AUC value was set to 1. The AUC value implies bioavailability of delivered drug through each delivery platform [43]. In surprise, bioavailability of FTA group was 93% which was comparable to bioavailability of injection group (100%) and 1.37-fold higher than that of DMN patch group (68%).

Our FTA system is platform for the DMN delivery, therefore any drugs, which are suitable for loading into the DMN can be candidate for our system. We demonstrated our FTA’s superior delivery efficacy than existing DMN patch with stable DMN formulation [44]. However, it will be needed to conduct skin safety test such as skin irritation and skin sensitive rate because DMN is delivered by transdermal route. In our further study, we believe that we can solve the issues we suggested.

We compared DMN insertion and drug delivery efficiency of FTA with those of the existing DMN patch through *in vivo* test. In addition, FTA group showed the maximum penetration of DMN inside the skin, even with only finger force (11.05 N) being applied compared with patch applicator’s spring elastic force (35.28 N). To sum up, the FTA system showed enhanced DMN drug delivery efficiency with application of only finger force *via* micropillars compared with that of the existing DMN patch. Thus, our FTA system is a novel and promising platform that can overcome the problem of insertion of DMN patch inside the skin.

## Conclusions

In this study, we successfully fabricated an applicator that work based on the novel concept of accumulated energy on polymer film through micropillars without any additional force application device; moreover, its application efficiency has been shown in an animal model using insulin as a model drug. By force analysis of CMC film, we ascertained that the film puncture force could be controlled by adjusting the concentration of polymer solution and diameter of micropillar. Since FTA showed successful and effective delivery of the protein drug insulin *in vivo*, we expect that FTA system may be finally utilized in delivering DMN-loaded drugs, such as vaccines, biosimilars, proteins, small molecules, steroids, and hormones, which are widely known drug candidates for microneedle application. In conclusion, our FTA system is a novel and promising platform and alternative to conventional DMN patch system with its improved productivity by its superior delivery rate and bioavailability; we suggest FTA as a new generation applicator for microneedle application in the future.

## Supplementary Information

**Additional file 1. **A zoomed image of single micropillar and DMN. DMN bottom diameter is larger than micropillar’s diameter (350 µm). The micropillar, punctured film, and CMN are indicated by arrowheads (Scale bar, 500 µm).

**Additional file 2. **Fluorescence intensity at the bottom of cadaver skin in the DMN patch and FTA groups. The value was expressed as relative fluorescence intensity of each group using the ImageJ program (*n* = 5, mean ± S.E.). The average fluorescence intensity of DMN patch was 70.36 ± 3.83 and FTA was 102.52 ± 7.81. Statistical significance was set at *p* < 0.05; ^*^p < 0.05, ^**^p < 0.01, ^***^p <0.001.

**Additional file 3. **(a) Blood glucose level expressed in a bar graph. (b) Plasma insulin level expressed in a bar graph. Statistical significance was set at *p* < 0.05; ^*^p < 0.05, ^**^p < 0.01, ^***^p < 0.001. n.s. indicates non-significant.

**Additional file 4. **Thickness of films prepared using 2%, 4%, and 6% CMC polymer solution (*n* = 10, mean ± S.E.). The thickness of films was 12.23 ± 1.20 µm, 23.05 ± 1.35 µm, and 33.14 ± 1.30 µm at 2%, 4%, and 6% CMC film concentrations, respectively.

## Data Availability

All data generated or analyzed during this study are included in this published article.

## Abbreviations

FTA: Film-trigger applicator
DMN: Dissolving microneedle
HA: Hyaluronic acid
CMC: Carboxymethyl cellulose
OCT: Optical coherence tomography
SEM: Scanning electron microscope
S.E.: Standard error
n.s.: Non-significant
