# Supplementary material for: Film-trigger applicator (FTA) for improved skin penetration of microneedle using punching force of carboxymethyl cellulose film acting as a microneedle applicator
Source: Biomater Res. 2022 Oct 5;26:53. doi: 10.1186/s40824-022-00302-5 (PMC9533547; doi:10.1186/s40824-022-00302-5)
Supplement: Supplementary file 1 — Additional file 1. A zoomed image of single micropillar and DMN. DMN bottom diameter is larger than micropillar’s diameter (350 µm). The micropillar, punctured film, and CMN are indicated by arrowheads (Scale bar, 500 µm). [file 40824_2022_302_MOESM1_ESM.docx]

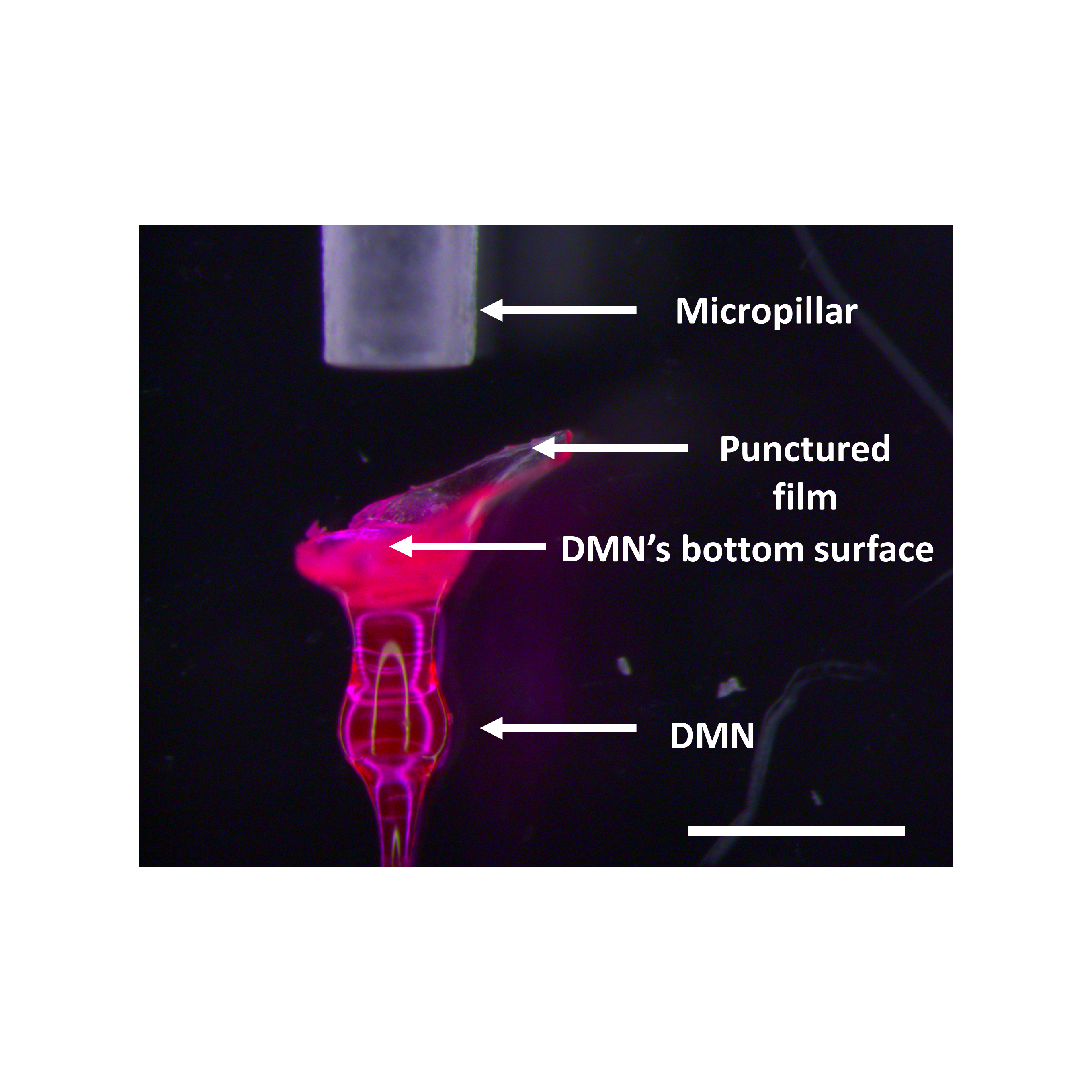


**Additional file 1.** A zoomed image of single micropillar and DMN. DMN bottom diameter is larger than micropillar’s diameter (350 µm). The micropillar, punctured film, and CMN are indicated by arrowheads (Scale bar, 500 µm).
