# Supplementary material for: Film-trigger applicator (FTA) for improved skin penetration of microneedle using punching force of carboxymethyl cellulose film acting as a microneedle applicator
Source: Biomater Res. 2022 Oct 5;26:53. doi: 10.1186/s40824-022-00302-5 (PMC9533547; doi:10.1186/s40824-022-00302-5)
Supplement: Supplementary file 2 — Additional file 2. Fluorescence intensity at the bottom of cadaver skin in the DMN patch and FTA groups. The value was expressed as relative fluorescence intensity of each group using the ImageJ program (n = 5, mean ± S.E.). The average fluorescence intensity of DMN patch was 70.36 ± 3.83 and FTA was 102.52 ± 7.81. Statistical significance was set at p < 0.05; *p < 0.05, **p < 0.01, ***p <0.001. [file 40824_2022_302_MOESM2_ESM.docx]

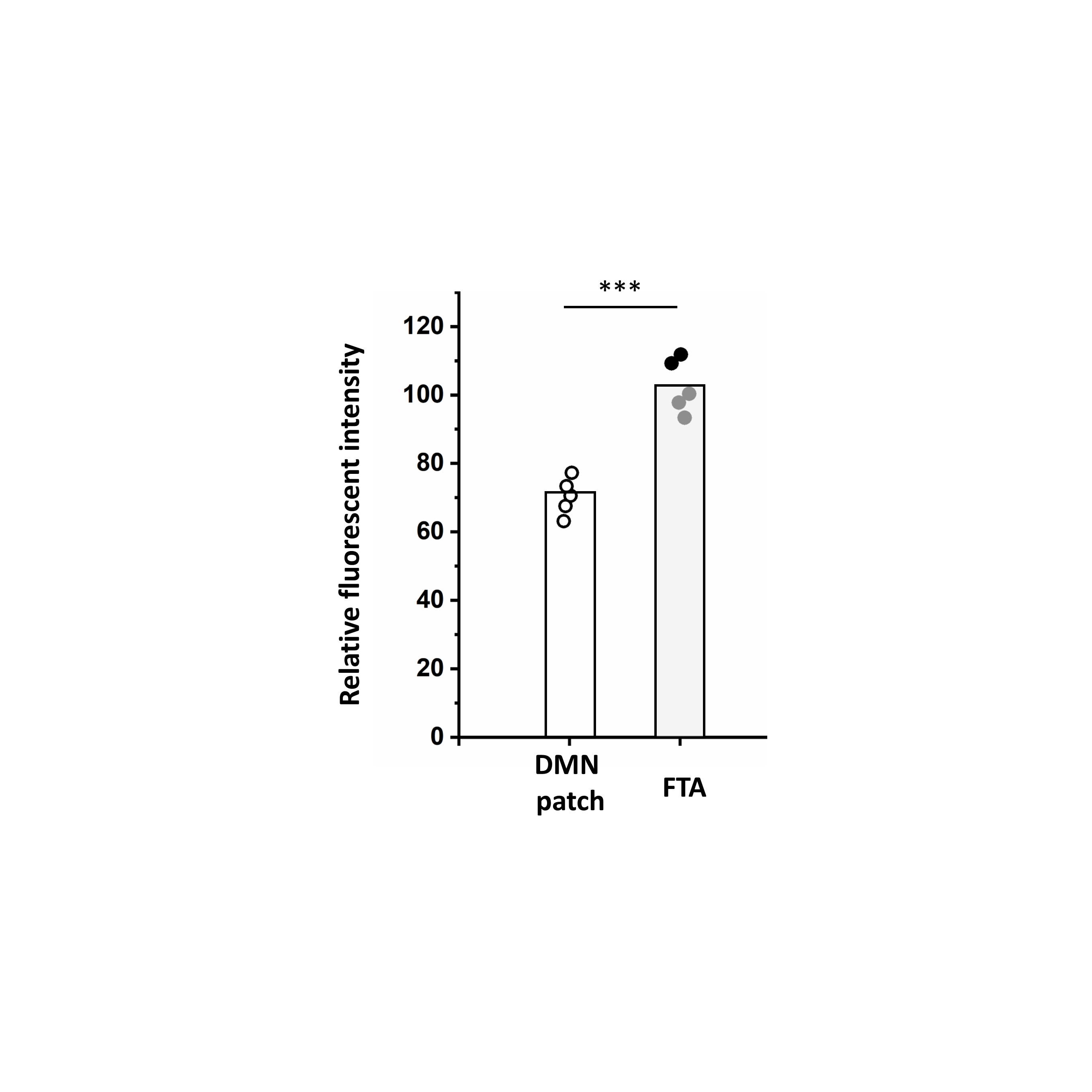


**Additional file 2.** Fluorescence intensity at the bottom of cadaver skin in the DMN patch and FTA groups. The value was expressed as relative fluorescence intensity of each group using the ImageJ program (n = 5, mean ± S.E.). The average fluorescence intensity of DMN patch was 70.36 ± 3.83 and FTA was 102.52 ± 7.81. Statistical significance was set at *p* < 0.05; ^*^*p* < 0.05, ^**^*p* < 0.01, ^***^*p* <0.001.
