# Supplementary material for: Film-trigger applicator (FTA) for improved skin penetration of microneedle using punching force of carboxymethyl cellulose film acting as a microneedle applicator
Source: Biomater Res. 2022 Oct 5;26:53. doi: 10.1186/s40824-022-00302-5 (PMC9533547; doi:10.1186/s40824-022-00302-5)
Supplement: Supplementary file 3 — Additional file 3. (a) Blood glucose level expressed in a bar graph. (b) Plasma insulin level expressed in a bar graph. Statistical significance was set at p < 0.05; *p < 0.05, **p < 0.01, ***p < 0.001. n.s. indicates non-significant. [file 40824_2022_302_MOESM3_ESM.docx]

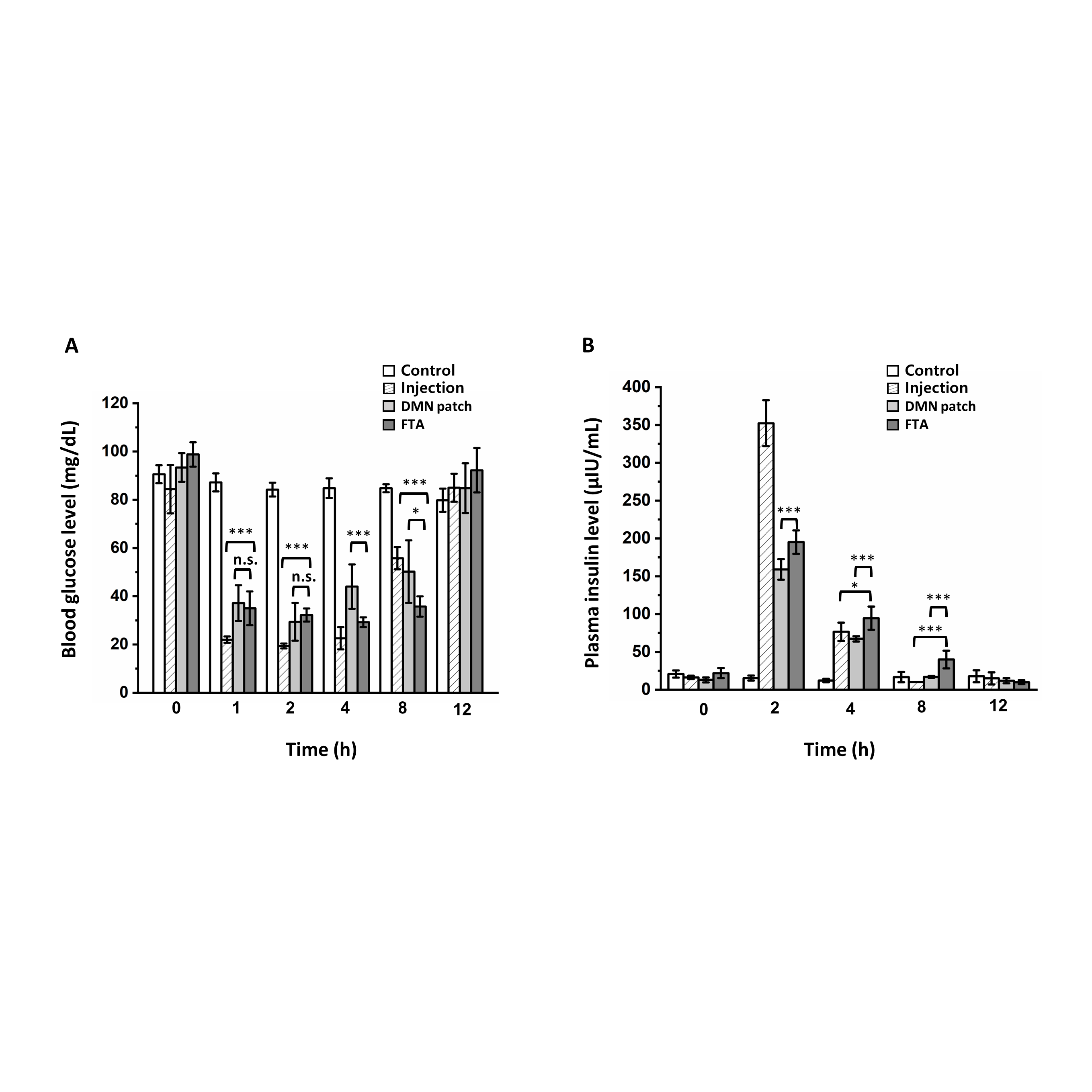


**Additional file 3.** **(a)** Blood glucose level expressed in a bar graph. **(b)** Plasma insulin level expressed in a bar graph. Statistical significance was set at *p* < 0.05; ^*^*p* < 0.05, ^**^*p* < 0.01, ^***^*p* < 0.001. n.s. indicates non-significant.
