# Supplementary material for: Film-trigger applicator (FTA) for improved skin penetration of microneedle using punching force of carboxymethyl cellulose film acting as a microneedle applicator
Source: Biomater Res. 2022 Oct 5;26:53. doi: 10.1186/s40824-022-00302-5 (PMC9533547; doi:10.1186/s40824-022-00302-5)
Supplement: Supplementary file 4 — Additional file 4. Thickness of films prepared using 2%, 4%, and 6% CMC polymer solution (n = 10, mean ± S.E.). The thickness of films was 12.23 ± 1.20 µm, 23.05 ± 1.35 µm, and 33.14 ± 1.30 µm at 2%, 4%, and 6% CMC film concentrations, respectively. [file 40824_2022_302_MOESM4_ESM.docx]

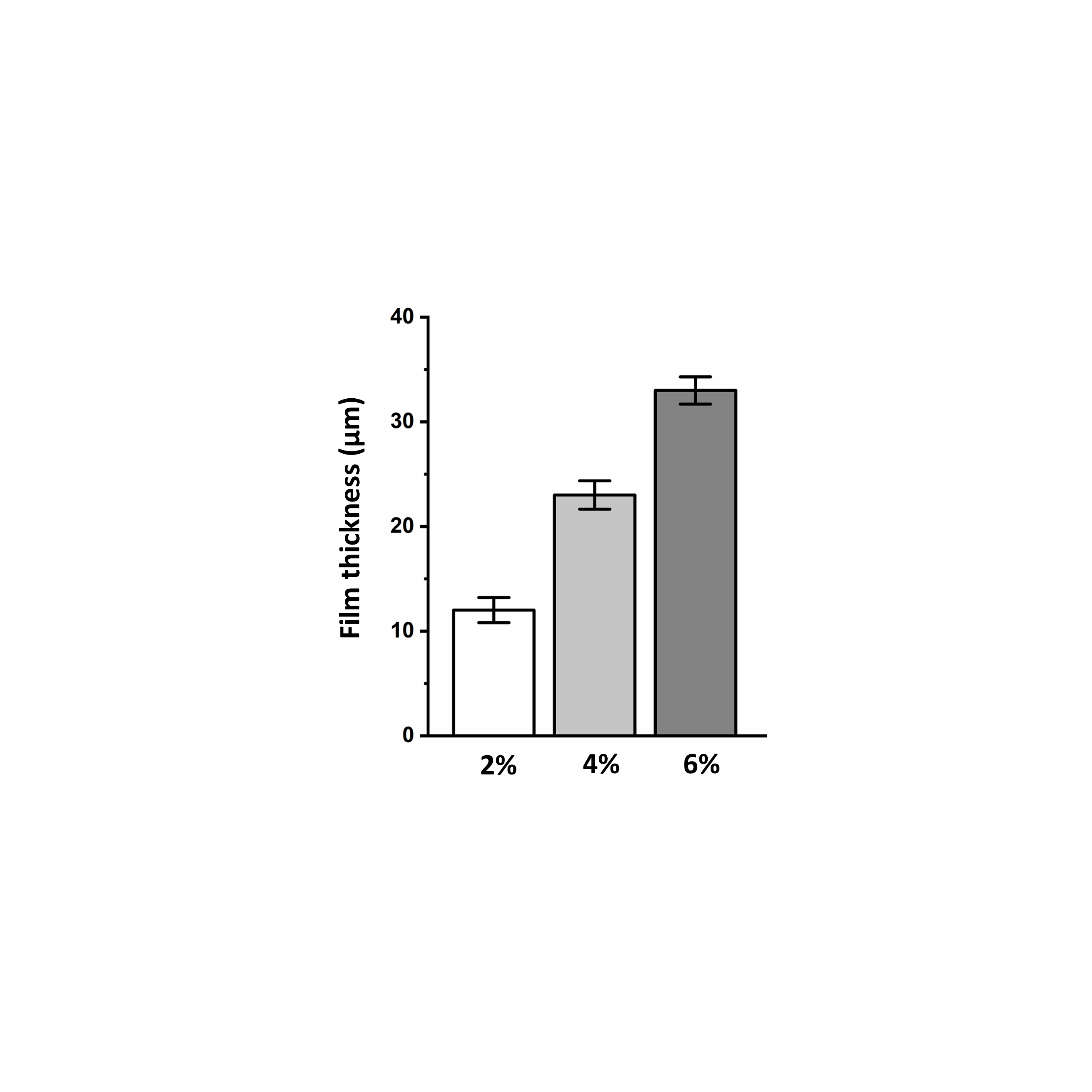


**Additional file 4.** Thickness of films prepared using 2%, 4%, and 6% CMC polymer solution (n = 10, mean ± S.E.). The thickness of films was 12.23 ± 1.20 µm, 23.05 ± 1.35 µm, and 33.14 ± 1.30 µm at 2%, 4%, and 6% CMC film concentrations, respectively.
